# The relationship between the nomophobic levels of higher education students in Ghana and academic achievement

**DOI:** 10.1371/journal.pone.0252880

**Published:** 2021-06-16

**Authors:** Harry Barton Essel, Dimitrios Vlachopoulos, Akosua Tachie-Menson

**Affiliations:** 1 Department of Educational Innovations in Science and Technology, Kwame Nkrumah University of Science and Technology, Kumasi, Ghana; 2 Faculty of Digital Media & Creative Industries, Amsterdam University of Applied Sciences, Amsterdam, Netherlands; International Medical University, MALAYSIA

## Abstract

There is an upsurge in the use of mobile phones among higher education students in Ghana, which may result in the nomophobia prevalence with the students. Therefore, the need to assess the influence of nomophobia within the student population in Ghana. This descriptive cross-sectional study investigated the prevalence of nomophobia and the sociodemographic variables, and the association with academic achievement of the understudied population. A self-reporting nomophobia questionnaire, composed of 20 dimensions, was answered by 670 university students to measure the nomophobia prevalence. Raw data were estimated using descriptive statistics, and one-way ANOVA and Independent T-test. While the findings showed diverse grades of nomophobia, statistical significance between academic achievement and the level of nomophobia was observed. This study concludes that there is a high nomophobia prevalence among university students in Ghana as the use of smartphones increases. However, follow-up studies should be conducted in Ghanaian universities to monitor nomophobia and its associates in order to reduce the adverse effects of habitual use of smartphones.

## Introduction

Smartphone technologies have revolutionised the way we communicate in barely a decade. According to Park [1], one of the globally preferred devices over a network is the smartphone, which is used to connect families and friends or to make inquiries. As manufacturers of smartphones continuously integrate advanced functionalities, the use of smartphones become more pervasive and an integral part of our daily lives [2–5]. The ease of portability [6], its high performance [7], and the extended possibilities smartphones offer [8,9] are influencing higher education students to engage in online activities such as social networking, online shopping [10], and gaming (online and offline) among others [11]. Besides, smartphones significantly influence humans in several aspects of life; initiate novel methods of executing tasks; and proffer innovative modes of sharing, garnering and processing data into information [1,12]. Diverse studies have confirmed that autonomous and excessive use of smartphone is prevalent [13,14] among higher education students considering their ability to manage and control accelerated technological advancements than other subpopulations [15–17]. Notwithstanding, excessive use of smartphones comes with some bottlenecks.

Alosaimi et al. [18] indicated that higher education students depend on their smartphones to get around the most obvious daily engagements making them waste a lot of time with their smartphones. Research has identified overexposure and inappropriate use of smartphones as some of the correlated challenges resulting from deficiencies in knowledge or education about smartphones [19,20]. Likewise, the extreme use of smartphones can result in total reliance and addictive behaviour [21–23] among higher education students. Besides, some negative consequences include increased anxiety and interference [24], reduced sense of volitional control [25] and increased psychological burnout [26]. Additionally, other studies have reported the influence excessive smartphone use has on the academic experience and achievement of students [27–29]. Current research [30,31] highlights the impact of the use of smartphones on academic success, since it was found that concentration on study, and practical work were negatively affected by smartphone dependency. Jena [32] accentuated that uncontrolled use of smartphones has myriad consequences resulting in unpredictable health concerns.

Research has shown that living without a smartphone or mobile phone have consequences on a person’s psychological health, and this led to the coining of a novel terminology nomophobia referring to smartphone separation anxieties [3,33–35], or a situational phobia where support is absent when experiencing an obnoxious circumstance [36]. The nomenclature nomophobia, formed of “No Mobile Phone Phobia”, defines the pathological anxiety with momentary detachment of a person from a mobile phone or smartphone, thus, either not having mobile networks or signal coverage [17], losing mobile phone contacts, or running out of battery power [37]. About 18.5–73.0 per cent of higher education students are vulnerable to severe nomophobia [37–40]. This condition is found to be associated with various sociodemographic factors such as gender, age, academic level, frequency of smartphone use, self-esteem, self-image, self-efficacy, extroversion, and impulsivity [39,41–48], as well as habits including alcohol consumption, smoking and duration of sleep [30]. Studies have evidenced a relationship between academic achievement and nomophobia behaviour [49] of higher education students. Several authors acknowledge that low academic productivity and achievement are associated with nomophobia [50–55]. Additionally, smartphone dependency negatively influences students’ involvement in practical activity, focus on studies and ultimately, academic performance [31].

Alas, there is scarcity of research assessing the levels and prevalence of nomophobia among higher education students in Ghana though there has been a notable upsurge of smartphone use in this population. Ghana is one of the frontliners in the adoption and statistically significant increases in Smartphone ownership [56]. Smartphone adoption in Ghana stands at 55% of the entire population, much higher than the regional average of 44.8% [57]. With the propagation and adoption of smartphones essentially by higher education students [2,33] and the adverse effect that nomophobia has on multivariable outcomes in other understudied populations [41], an inquiry into nomophobia prevalence among higher education students in Ghana will lead to a complete awareness of how the smartphone technologies are affecting higher education students in Ghana. This study, therefore, examines the nomophobia prevalence, the sociodemographic variables influencing nomophobia index, as well as the associations between nomophobia and academic achievement among higher education students in Ghana. The outcome of this study on nomophobia will offer sufficient awareness about this population to policy makers of higher education institutions in Ghana towards the advancement of educational policies and feasible therapeutic solutions at diverse phases of prevention.

The following research questions (RQ) drive the study:

**RQ 1**
  What is the prevalence of nomophobia with Kwame Nkrumah University of Science and Technology (KNUST) students?
**RQ 2**
  Do the variations in selected sociodemographic variables influence the frequency of nomophobic incidence with students in KNUST?
**RQ 3**
  Is the association between the nomophobic index and academic achievement statistically significant with students in KNUST?

## Materials and methods

### Research design, research area and sample groups

This current study applied the quantitative method of research, particularly the descriptive, transversal, and correlation research designs. The cross-sectional design was considered the most adequate methodological decision, since it used to examine a population at a single point in time, like taking a cross-section of a group, and variables are recorded for each participant [46,50,58]. According to Gravetter & Forzano [59], cross-sectional designs are adequate for answering research questions related to the incidence or prevalence of a condition, belief or situation. All the participants included in the present study were selected across the six (6) Colleges in KNUST. The university is located in the West African region of the African continent, and it is reputed as one of the best in the region. The 6 colleges are 1) Art and Built Environment 2) Health Science 3) Sciences 4) Engineering 5) Agriculture and Natural Resources, and 6) Humanities and Social Sciences. The population constituted undergraduates students who registered for the second semester of the 2019/2020 academic year. The students were included provided they were above 18 years old, of any gender, who wanted to participate in the study voluntarily [60]. The current study was conducted between April and September 2020. The data collection took place between April and July 2020.

### Sampling technique and sample size

The population of undergraduate students in KNUST is more than 10,000. We used Yamane’s sample size determination formula (1973) to estimate the target students for the current study. The Yamane’s formula is illustrated as:

$$n=\frac{N}{1+N\left(e\right)2}$$

***n*** = size of sample,

***N*** = size of population,

***e*** = the acceptable error margin (95% confidence level; p = .5 assumed).

According to the formula, the minimum sample for the population understudied should be *n* = 385. We retrieved a total of 683 eQuestionnaires after the online data collection process ended on 10th September 2020. No eQuestionnaire with missing data was recorded. However, some students were excluded (*n* = 13) on the basis of providing extreme scores (Mahalanobis Distances of p < .001). After the data wrangling and cleansing, the final sample derived for the current study was *n* = 670 students. The investigators used the convenience sampling approach to choose the students for the current study.

With the *n* = 670 students who partook in the current study, 397 (59.3%) were females and 273 (40.7%) were males. The ages of the students ranged between 18 years and 29 years with an average age of 21.3 and standard deviation SD = 1.91. Table 1 shows the distribution of sociodemographic profiles of the students. All the sampled students reported ownership of a smartphone.

**Table 1:** Distribution of sociodemographic profiles of the participants.

Variables		M	SD	Frequency (%)
Gender	Male			273 (40.7)
	Female			397 (59.3)
Age		21.3	1.9	
Academic level	Year One			296 (44.2)
	Year Two			143 (21.3)
	Year Three			127 (19.0)
	Year Four			104 (15.5)
Residential Status	On-Campus			310 (46.3)
	Off-Campus			360 (53.7)
Marital status	Married			101 (15.1)
	Single			468 (69.9)
	Divorced			18 (2.7)
	In a relationship			83 (12.3)
Hours of smartphone usage per day		7.2	3.9	
Times per day checking smartphone		39.1	41.4	
Time of the day phone use is more	Day			224 (43.5)
	Night			446 (66.5)
Active internet service	Yes			661 (98.7)
	No			9 (1.3)
Daily internet usage time		4.21	2.05	
Number of Phones	1			597 (89.1)
	≥ 2			73 (10.9)
College/ Academic Discipline	Health Science			103 (15.9)
	Science			112 (17.3)
	Art and Built Environment			116 (17.9)
	Engineering			106 (16.4)
	Agriculture and Natural Resources			101 (15.5)
	Humanities and Social Sciences			110 (17.0)

### Data collection tools and procedure

#### Nomophobia Questionnaire (NMP-Q)

The self-reporting NMP-Q Likert scale, composed of 20 dimensions and validated by Yildirim and Correia [34], was used to collect data from the students. The scale measures nomophobic behaviour (fear of separation from one’s phone) with four latent constructs (see Table 2). Each dimension in a construct is estimated on a Likert scale of seven pointers, with pointer 1 as “strongly disagree” and pointer 7 as “strongly agree”. The dimensions were coded to gain a clear understanding of the results. The NMP-Q scale provides a numerical score varying from 20 to 140, with maximum scores (NMP-Q = 140) symbolising highest severity [41] of nomophobic behaviour. A score between 100 and 140 infer severe nomophobia, scores between 60–99 indicate that nomophobia is moderate, scores 21–59 infer that nomophobia is mild, and a score of 20 infers that nomophobia is absent [34,60,61]. Also, it is important to mention that the Kaiser-Meyer-Olkin (KMO) measurement [62] and Bartlett’s test of sphericity [63] were computed to estimate the sampling adequacy for factorability. The dataset garnered was accepted for a PCA based on the assumptions that the KMO was > 0.60, and the Bartlett test of sphericity was less than P-value (p < .05).

**Table 2:** The constructs of the NMP-Q scale and corresponding number of dimensions.

Number	Latent Constructs	Code	Number of dimensions
1	Not being able to communicate	NMPQ_C01	6
2	Losing connectedness	NMPQ_L02	5
3	Not being able to access information	NMPQ_I03	4
4	Giving up convenience	NMPQ_G04	5
Total			20

NMPQ: Nomophobia Questionnaire.

The seminal study with the NMP-Q scale [34] generated a Cronbach’s alpha coefficient of *α* = .945, indicating excellent internal consistency and construct validity (r = .710). The current study reported an overall Cronbach’s alpha (and, McDonald’s omega) of *α* = .97 (ω_t_ = .97) which is excellent, and for each of the constructs: construct 1, unable to communicate (α = .93, ω_t_ = .94); construct 2, losing connectivity (α = .88, ω_t_ = 0.89); construct 3, unable to reach to information (α = .93, ω_t_ = .93); and construct 4, giving up convenience (α = .95, ω_t_ = .95).

Graphical inspection (Q-Q plot and Histogram) and the Shapiro-Wilk test (p>.05) confirmed that the NMP-Q scores were normally distributed. Consequently, we obtained satisfactory skewness and kurtosis values of ±1.96, indicating normality of distribution for the NMP-Q scores [64,65].

The investigators facilitated the collection of data from the students by customising NMP-Q scale into an e-Questionnaire with Google Forms application. Additionally, a single-blind data collection was employed so that the students would not be in the know regarding the aims of the study. The investigators adopted this process in an attempt not to generate social desirability, reactivity and expectations [19] among the students. The e-Questionnaire was distributed by generating a short URL or link which was forwarded to the students via their institutional electronic mail (email) and Short Message Service (SMS). Students were restricted to submit one response in the online survey system.

#### Data management and analysis

For data computations and analyses, the investigators used Jamovi open-source statistical package, version 1.6.22. To address RQ 1, we represented the distribution of the sociodemographic variables (Gender, Age, Marital Status, Academic grade, Residential Status, College/Academic discipline, and Smartphone use) with frequencies and percentages. Means (*M*), standard deviations (*SD*), skewness and kurtosis, and standard error of measurement (SEM) on the data garnered from the NMP-Q scale to estimate the nomophobia prevalence among the students. To address RQ 2, we applied one-way analysis of variance (ANOVA) and independent samples T-test to estimate the statistically significant differences between the sociodemographic variables and the NMP-Q scores. In RQ 3, we applied a Chi-square (*X*^*2*^) Test of Independence to determine whether the relationship between the nomophobia prevalence and academic achievement (CWA) was statistical significance. The level of statistical significance set for this current study was α = .05 (2-tailed) with a confidence interval of 95%.

#### Measurement of academic achievement

The academic achievement of students was measured using the current Cumulative Weighted Average (CWA) of the academic year (2019/2020). The CWA is taken as a continuous variable. The investigators adopted the KNUST standard categorisation system (division) to grade the achievement (CWA) of the students. The CWA categorisation system is as follows: First class division (100.0–70.0), Second Upper class division(69.9–60.0), Second Lower division (59.9–50.0), and Pass division (49.9–40.0).

### Ethical consideration and clearance

The current study was approved by the Centre of Excellence for Learning, Teaching and Research (CELTR) of the Faculty of Educational Studies Academic (FES-CELTR reference number: FES-CELTR/221/08/2020) at KNUST, Ghana. All the participating students gave digitally informed consent indicated by clicking the "I Agree" button before proceeding to the first item on the eQuestionnaire. The students were assured of absolute confidentiality and anonymity of responses they provide for the study.

The study conforms to the Declaration of Helsinki (1964) with its succeeding revisions on ethical criteria. The researchers also sought consent from the developers of the original 20-item NMP-Q scale (Yildirim and Correia), who in turn, approved the use of the scale. No changes were made to the wording of the original instrument.

## Results

### Nomophobia prevalence among KNUST students

**Fig 1:**
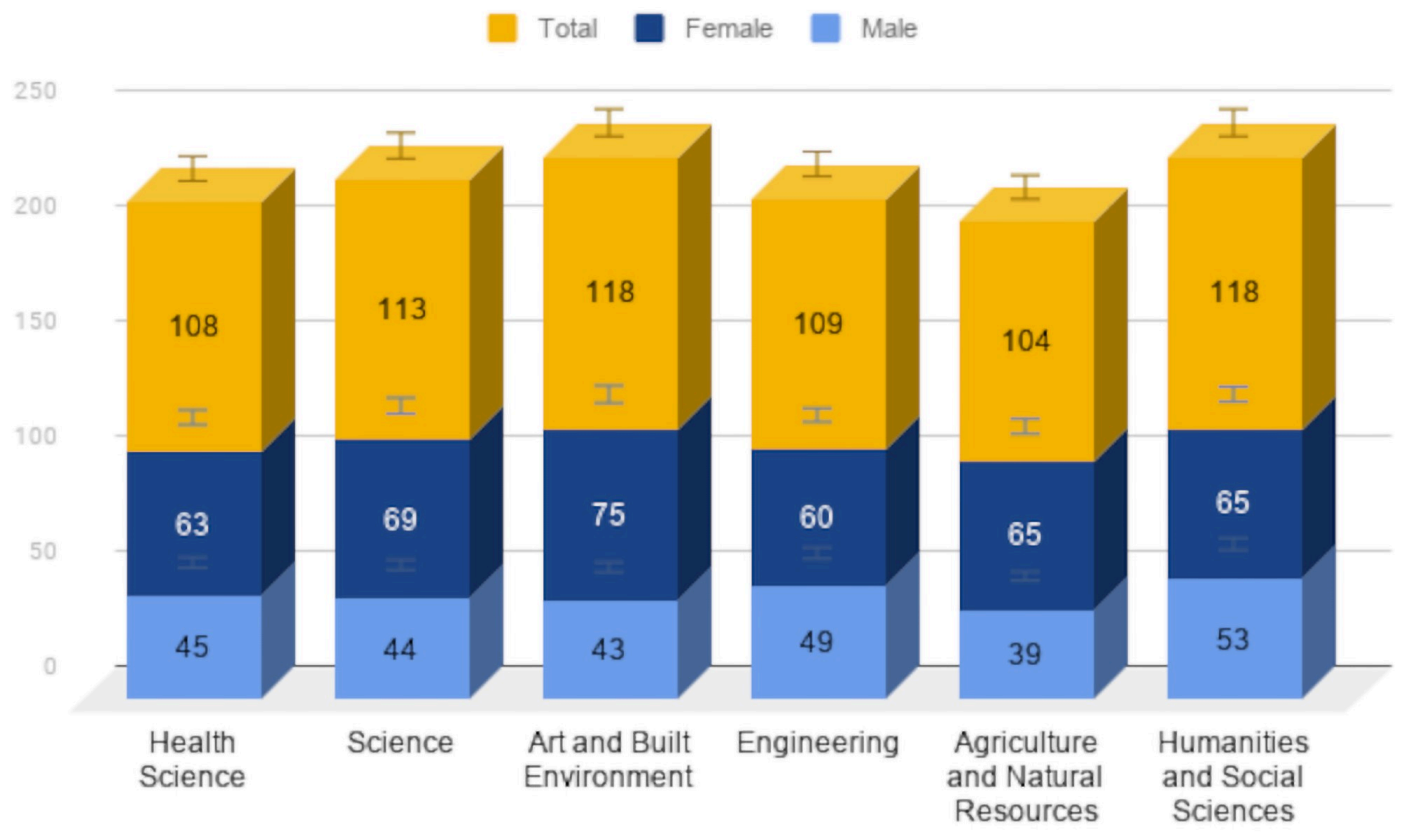
Gender distribution in the six colleges.

To estimate nomophobia prevalence among the students, we applied the 20-item NMP-Q scale developed [34] to collect data from a sample of *n = 670* students as discussed in the methods section. Fig 1 illustrates gender distribution across the six colleges in the Kwame Nkrumah University of Science and Technology.

**Fig 2:**
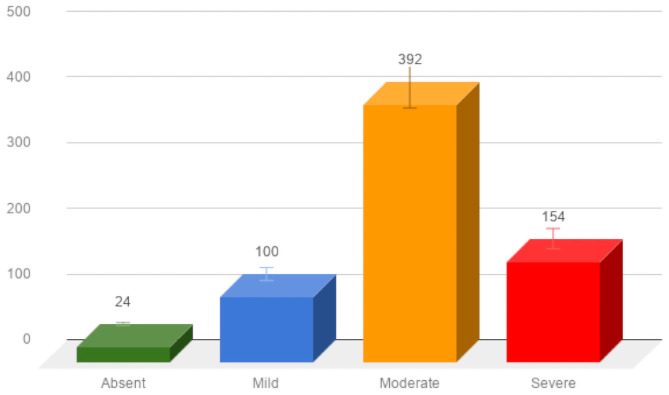
Distribution of student across the four nomophobia categories.

**Fig 3:**
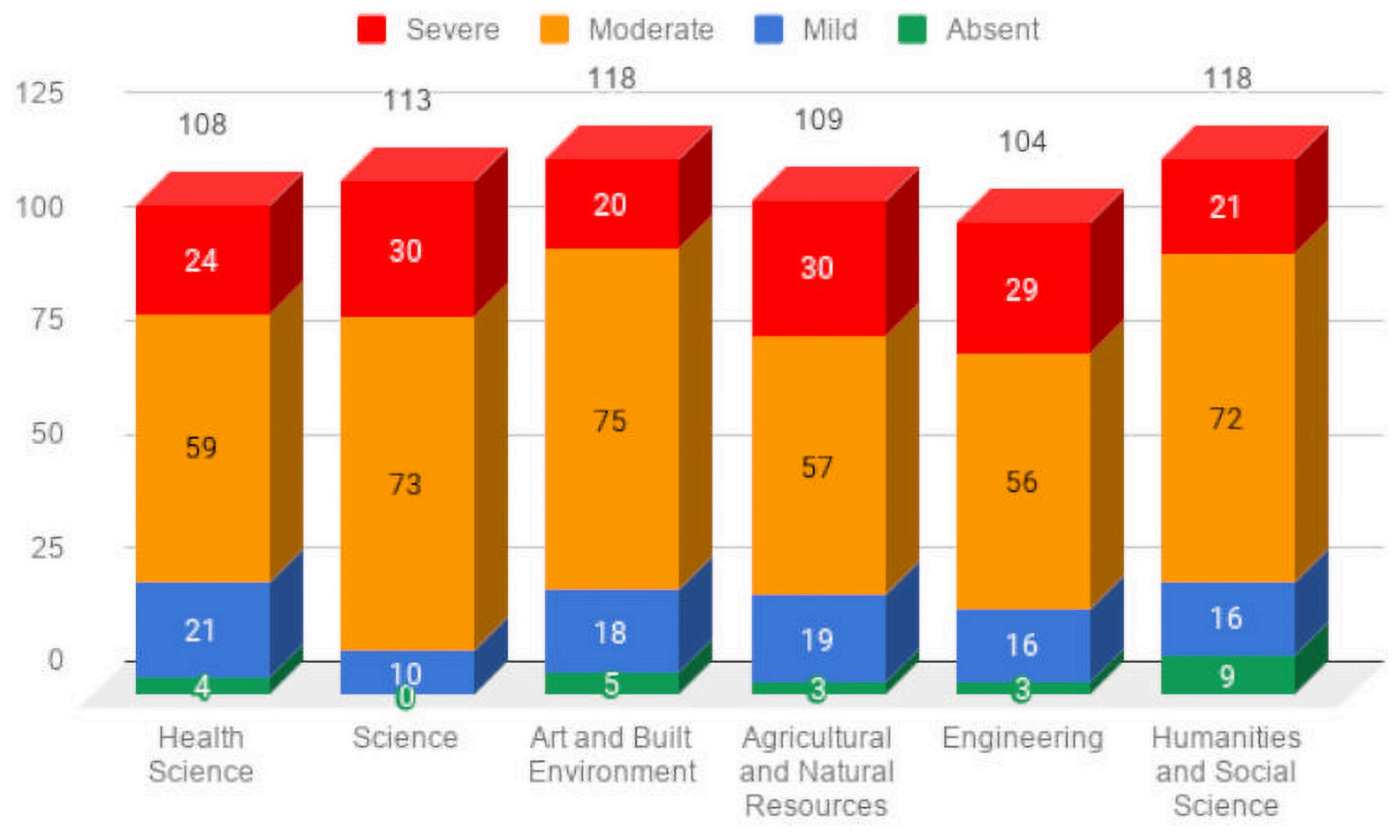
Academic disciplines and the levels of nomophobia prevalence.

Table 3 shows the representative statistics of the nomophobia questionnaire scores of the students understudied. The data demonstrate that 3.6% (24/670) of students reported the absence of nomophobia; notwithstanding, 14.9% (100/670), 58.5% (392/670), and 23.0% (154/670) reported mild, moderate, and severe nomophobia, accordingly. Nomophobia prevalence among the students understudied was estimated at 96.4% (646/670). The total NMP-Q items had a mean score of m = 83.8 (SD = 24.0, range: 20–135), giving a clear indication of a moderate prevalence of nomophobia among the students. We found no statistically significant difference (p = 0.057) between Colleges/Disciplines and the nomophobia score (see Table 4). Additionally, Figs 2 and 3 illustrate the distribution of nomophobia among the students, and the levels of nomophobia prevalence within the disciplines accordingly.

**Table 3:** Descriptive statistics of the levels of NMP-Q.

Factors	M	SD	Frequency (%)	Reliability (α, ω_t_)
Level of Nomophobia				
Absence (≤ 20)			24 (3.6)	
Mild (21–59)			100 (14.9)	
Moderate (60–99)			392 (58.5)	
Severe (100 ≥)			154 (23.0)	
Total score of NMP-Q	81.8	26.2		0.97, 0.97

NMP-Q: Nomophobia Questionnaire.

**Table 4:** Nomophobia scores for colleges/disciplines (N = 670).

College/Discipline	N	NMP-Q score	SD	*p*-value
Health Sciences	108	80.7	27.2	0.057
Science	113	86.5	20.1	
Art and Built Environment	118	78.2	25.7	
Agriculture and Natural Resources	109	82.4	29.1	
Engineering	104	85.1	27.4	
Humanities and Social Sciences	118	77.7	26.1	
Mean NMP-Q score		81.8		

NMP-Q: Nomophobia Questionnaire; M: Mean; SD: Standard Deviation.

From the analysis in Table 5, it is evident that the constructs “Not being able to communicate”, “Losing connectedness” and “Giving up convenience” had a mean greater than the average NMP-Q mean score; however, the constructs “Not being able to access information” was less than the mean score (81.8/4) of the NMP-Q scale. Besides, "Not being able to communicate" of the nomophobia constructs had the highest total average score (M = 24.5, SD = 7.82) while the lowest total average score of the constructs is " Not being able to access information" (M = 17.6, SD = 5.19).

**Table 5:** Descriptive statistics of students’ scores with the NMP-Q scale (*N* = 670).

Scale	Constructs	N	M	SD	SEM	Skewness	Kurtosis
1	Not being able to communicate	670	23.9	7.82	0.607	-0.329	0.611
2	Losing connectedness	670	20.5	5.97	0.635	0.069	0.698
3	Not being able to access information	670	17.2	5.19	0.704	0.099	0.759
4	Giving up convenience	670	20.1	7.40	0.2791	0.634	-0.787
	NMP-Q	670	81.8	24.0	0.944	-0.245	0.665

NMP-Q: Nomophobia Questionnaire.

In Table 6, the data demonstrate the most predominantly smartphone applications used by the students. Whatsapp (n = 242; 39.2%) is leading the list, confirming the current literature on this topic [66], since it allows users to send unlimited texts and it is affordable because students only incur costs of Internet use; Twitter (n = 210; 35.9%), instagram (n = 131; 33.3%), and YouTube (n = 85; 30.8%) were the most frequently used applications, representing the first, second, third and fourth most used smartphone applications, respectively. Additionally, Table 5 shows that a percentage of the students also used TikTok (n = 32; 5.2%), Facebook (n = 20; 3.2%), and Games (n = 19; 3.1%).

**Table 6:** Frequently used smartphone applications (*N* = 670).

Application	First, n (%)	Second, n (%)	Third, n (%)	Fourth, n (%)
Whatsapp	242 (39.2)	192 (32.8)	77 (19.5)	53 (19)
Twitter	188 (30.4)	210 (35.9)	91 (23.1)	38 (3.6)
Instagram	65 (10.5)	73 (12.5)	131 (33.3)	19 (6.8)
YouTube	52 (8.4)	41 (7)	39 (9.9)	85 (30.8)
Tiktok	32 (5.2)	33 (5.6)	25 (6.4)	33 (11.8)
Facebook	20 (3.2)	21 (3.6)	20 (5.1)	25 (9)
Games	19 (3.1)	15 (2.6)	11 (2.8)	13 (4.7)

### Selected sociodemographic variables and their relationship with the nomophobia scores

ANOVA and independent samples t-tests were estimated to associate selected sociodemographic variables with the average NMP-Q score. The nomophobia (NMP-Q) score across selected sociodemographic profiles. The study found no statistically significant difference between nomophobia scores and academic level [F(3, 644) = 1.120, *p* = 0.342], residential status [*t*(646) = -1.120; *p* = 0.306], marital status [*t*(646) = -0.345; *p* = 0.731), and the colleges [*F*(5, 642) = 2.16; *p* = 0.057], as illustrated in Table 7.

**Table 7:** Distribution of sociodemographic profiles of the students (*N = 670*) and their nomophobia scores.

Variables		Frequency (%)	NMP-Q Score (Mean ± Standard Deviation)	*t/F*	*P*-value
Gender	Male	251 (38.7)	78.8.0 ±28.8	0.156	0.876
	Female	397 (61.3)	83.7 ±24.0		
Academic level	Year One/Level 100	294 (45.4)	82.1 ±24.6	1.120	0.342
	Year Two/Level 200	133 (20.6)	85.8 ±24.2		
	Year Three/Level 300	120 (18.5)	86.0 ±24.6		
	Year Four/Level 400	101 (15.6)	82.5 ±20.7		
Residential Status	On-Campus	294 (45.4)	82.9 ±24.0	-1.020	0.306
	Off-Campus	354 (54.6)	84.8 ±24.0		
Marital status	Married	101 (15.6)	83.6 ±23.7	-0.345	0.731
	Not Married	547 (84.4)	84.5 ±25.4		
College/ Academic Discipline	Health Sciences	103 (15.9)	83.0 ±29.4	1.330	0.250
	Science	112 (17.3)	86.5 ±20.1		
	Art and Built Environment	116 (17.9)	80.3 ±23.7		
	Engineering	106 (16.4)	84.2 ±27.5		
	Agriculture and Natural Resources	101 (15.5)	87.1 ±25.4		
	Humanities and Social Sciences	110 (17.0)	81.9 ±21.7		

Although no significant variation was found in the academic years of students, the level 100 students reported the lowest nomophobia score (*m = 82*.*1*, *SD = 24*.*6*). Concerning gender, males reported marginally high nomophobia score (*m = 84*.*0; SD = 23*.*9*). Students in the College of Humanities and Social Science had the lowest average NMP-Q scores (m = 81.9; SD = 21.7), while those in Agriculture and Natural Resources had the highest score (*m = 87*.*1*, *SD = 25*.*4*) among the six colleges.

Concerning residents in a room, the present study found no significant difference in the nomophobia scores of students who were living alone and those with roommates (*p* = 0.694) in the hostels (off-campus or on-campus). Notwithstanding, students who were single occupant had the highest average NMP-Q score (*m = 88*.*7; SD = 24*.*7*).

Regard course trailings (referrals), the present study did not find significant variations (*p = 0*.*876*) in nomophobia among students who had course referrals and those who did not have any course referrals. However, students with course referrals recorded the highest average nomophobia score *(m = 89*.*9 SD = 30*.*3*). The higher variation (*SD = 29*.*9*) among students with course referrals reflects the no significant statistical value recorded.

No statistically significant variation (*p = 0*.*186*) in nomophobia was identified concerning the religious affiliations of students. Though students in other religions reported the least average NMP-Q score (*m = 78*.*9; SD = 24*.*0*), students who are Christians reported the highest average NMP-Q score (*m = 88*.*1; SD = 24*.*7*) in the present study.

No statistically significant variation (*p = 0*.*235*) in nomophobia was reported among students who participated in extracurricular activities (sports, hall or hostel administration, debate, or politics) and those who did not engage in extracurricular activities. Notwithstanding, the average nomophobia score (*m = 86*.*9; SD = 24*.*7*) was higher among students who participate in extracurricular activities.

Concerning students who had repeated an academic year once or not, the study found no significant statistical difference (*p = 0*.*198*) in nomophobia. However, the average nomophobia score (*m = 83*.*4; SD = 23*.*5*) was higher with students who have never repeated an academic year.

The nomophobia score was not statistically significant (p = 0.235) among students’ habits such as sleep duration (*p* = 0.891) and eating (*p* = 0.557). Notwithstanding, students who enjoy eating a lot reported a higher average NMP-Q score (*m = 87*.*6; SD = 23*.*6*) compared to others.

### Association between nomophobia levels and academic achievement of students

**Fig 4:**
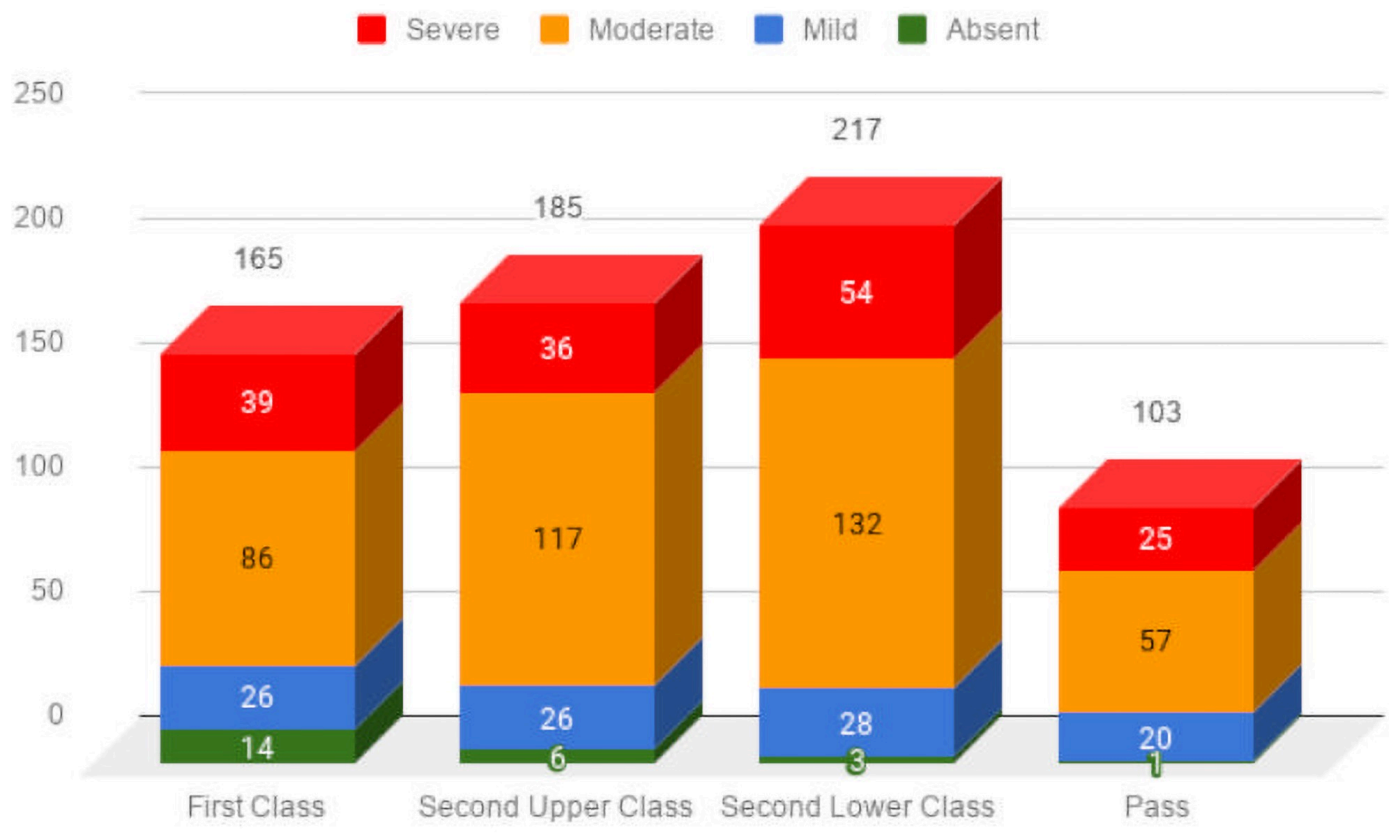
Visualisation of levels of nomophobia and academic achievement.

A Chi-square test of independence was performed to estimate the relationship between the levels of nomophobia and students’ academic achievement. The results indicated that the association between the variables is statistically significant, *X*^*2*^*(9*, *N = 670)* = 21.9, *p* = 0.009. The test thus shows that differences in academic achievement according to the different levels of nomophobia are significant. The adjusted standardized residual of 3.2 (58%) for first class achievement of students with absence of nomophobia contributed to the significant relationship, showing that the absence of nomophobia is associated with better academic achievement. The standardized residual was tested against a Bonferroni adjusted alpha level of p < 0.003 (0.05/16). The Table 8 shows the tabulation of the results from the Chi-Square analysis (*N* = 670). Additionally, Fig 4 visualises the interaction in each category of academic achievement and the level of nomophobia.

**Table 8:** Levels of nomophobia prevalence and academic achievement (*N = 670*).

Level of Nomophobia	Academic Achievement			
	First Class (%)	Second Upper Class (%)	Second Low Class (%)	Pass (%)
Absent	14 (58)	6 (25)	3 (12.5)	1 (4.2)
Mild	26 (26)	26 (26)	28 (28)	20 (20)
Moderate	86 (21.9)	117 (29.8)	132 (33.7)	57 (14.5)
Severe	39 (25.6)	36 (23.4)	54 (35.1)	25 (16.2)

## Discussion

Several studies discuss nomophobia prevalence among university students as a global concern. However, there is a dearth of inquiries addressing the same challenge in Africa, especially Ghana. This study, to our knowledge, is the first study evaluating the prevalence of nomophobia, sociodemographic variables and association with academic achievement of students in an institution of higher learning (KNUST) in Ghana. Besides, this subpopulation is the first to be investigated regarding the prevalence of nomophobia in Ghana.

The prevalence of nomophobia among distinct populations, notably adolescents [67], the youth [19] and higher education students [9,50,58], has been evidenced in several studies. Besides, studies have demonstrated that young adults (university students) routinely experience the highest nomophobia prevalence; and, the prevalence ranges between 77 percent and 99 percent among emerging and developed nations [44,68]. In the current study, we identified nomophobia prevalence of 96.7% with higher education students. Nomophobia prevalence was high among females (99.7%), on-campus students (98.3%), and the science discipline (100%). The statistic shows that the entire sample demonstrated some grade of nomophobia, with the majority (58.5%) of the students manifesting moderate nomophobic behaviour. Several studies [30,41,43] have reported a moderate nomophobia level among higher education students which align with the current study.

Notably, the outcomes of the current study demonstrate that the principal anxiety of students is exhibited on the construct "Not being able to communicate" as the construct received the largest scores. This finding supports the study by Moreno-Guerrero et al. [19], and other studies [15,17,69–73] carried out on similar population. In their studies, they found that "Not being able to communicate" garnered the highest scores among the four constructs. Moreno-Guerrero et al. [19], as well as Betoncu and Ozdamli [14], Ramos-Soler et al. [74], Kim, LaRose and Peng [75], and Gutiérrez-Puertas et al. [76], proclaimed that the failure to communicate or reach family or friends rapidly is one of the main factors that bring the feeling of uneasiness among higher education students. The finding is significant for educational policymakers to monitor further the psychological indications of smartphone use and ownership [2,41], among other subpopulations in Ghana.

Regarding the relative impact of sociodemographic variables on nomophobia, no statistically significant variations between gender and the smartphone use were found. Accordingly, it was observed in the current study that female participation was transcendent, which is in line with the research where females constituted a larger sample of the entire participants [41,77,78]. Hence, the females in the current study were found to have higher levels of nomophobia than males. Concerning the academic discipline, students in the College of Science and the College of Agriculture and Natural Resources demonstrated higher grades of nomophobic behaviour.

Interestingly, we observed no significant variation between students who were resident on campus and those who were not. The finding may be related to the "in-out-out-out" policy of residence instituted by the Kwame Nkrumah University of Science and Technology where only first years reside on campus. Qutishat et al. [30] and Madhusudan [45], in their study, found related results. Importantly, the present study found no significant variation between nomophobia and student habits such as sleep duration and eating behaviour, supporting the work of Qutishat et al. [30] in which differences in student sleep duration was not statistically significant.

When the relative effect of nomophobia on academic achievement was examined, we found a significant effect between high academic achievement and the absence of nomophobia. There are similar studies that reported the relationship between nomophobia and academic achievement [79,80]. Nonetheless, the outcome of the current study goes contrary to the study conducted by Qutishat et al. [30]. In their study, there was no significant correlation between academic achievement and nomophobia; however, they identified a marginal relationship in the achievement of students with severe nomophobia which explicated no statistical significance. Additionally, Ahmed et al. [50] reported a related outcome which suggested an inverse association among students’ performance and nomophobia but with no statistically significant difference. The difference between the current study and work of Qutishat et al. and Ahmed et al. may be attributable to the fact that our study was sited in a public university and was not discipline-specific.

Subsequently, this study was presented, bearing in mind some methodological constraints. We engage only higher education students in a particular public university in Ghana (KNUST), which makes it challenging to hypothesise the outcome of the current study for both private and public universities and the diverse socio-economic communities in Ghana. The study employed the convenience sampling technique to recruit the students that might have interjected some measure of selection bias in the study. Additionally, some degree of subjectivity was anticipated in the analysis of students’ perceptions using a non-experimental design. But it would be appropriate to advance studies in this direction by including and examining other sociodemographic variables as predictors and extend the studies to include larger subpopulations, as we confront a rapidly emerging and problematic social issue.

## Conclusion

Nomophobia or mobile phone separation anxiety, has become commonplace in today’s world where the use of smartphones play an important part in the wellbeing of people and their adaptation to their environment. We observed no significant gender difference concerning nomophobia prevalence. The current study has demonstrated the preliminary evidence for the predictors of nomophobia (96.7%) with university students in Ghana, especially among females. Furthermore, it has shown moderate to severe levels of nomophobia prevalence, highlighting the likely consequence of these measures of nomophobia on the academic success of students. Finally, the current study indicates that the significance of examining the prevalence of nomophobia and other correlated sociodemographic variables that would be beneficial for further studies in this area of research to proffer satisfactory protective programs and initiate therapeutic strategies for the subpopulations with this risk via awareness campaigns.

Academic administrators and policymakers in the university should be the front liners to ensure effective implementation of smartphone use policy for learning and teaching, and during students’ stay on campus. Further research should be conducted to extend knowledge on the effect of the increasing usage of smartphones among this population.
